# Association of programmed death ligand 1 expression with prognosis among patients with ten uncommon advanced cancers

**DOI:** 10.2144/fsoa-2020-0063

**Published:** 2020-08-19

**Authors:** Torben Steiniche, Morten Ladekarl, Jeanette Bæhr Georgsen, Simon Andreasen, Michael Busch-Sørensen, Wei Zhou, Matthew J Marton, Scott K Pruitt, Fan Jin, Kai-Li Liaw

**Affiliations:** 1 Institute of Pathology, Aarhus University Hospital, Aarhus DK-8200, Denmark; 2 Department of Oncology, Clinical Cancer Research Center, Aalborg University Hospital, Aalborg, Denmark; 3 Department of Otorhinolaryngology, Head & Neck Surgery & Audiology, Copenhagen University Hospital, Copenhagen, Denmark; 4 Department of Otorhinolaryngology & Maxillofacial Surgery, Zealand University Hospital, Køge, Denmark; 5 Busch-Sørensen Consulting, Virum, Denmark; 6 Merck & Co., Inc., Kenilworth, NJ, USA

**Keywords:** anal carcinoma, biliary adenocarcinoma, cervical carcinoma, endometrial carcinoma, mesothelioma, neuroendocrine tumors, salivary gland carcinoma, small-cell lung carcinoma, thyroid carcinoma, vulvar carcinoma

## Abstract

**Aim:**

PD-L1 expression and high levels of microsatellite instability (MSI-H) may predict response to checkpoint inhibitors, but their prevalence and prognostic value are unknown in many cancers.

**Methods:**

We retrospectively evaluated PD-L1 combined positive score (CPS) and MSI-H and their association with clinical outcomes among patients with ten advanced uncommon cancers.

**Results:**

398 of 426 patients (93%) had a valid PD-L1 result; most (242; 61%) had CPS ≥1. Prevalence of MSI-H tumors was 8/360. Median overall survival was shorter among patients with PD-L1 CPS ≥1 tumors after first-line treatment (23.0 vs 39.7 months, p = 0.014).

**Conclusion:**

PD-L1 was commonly expressed in solid tumors, and CPS ≥1 was associated with shorter overall survival. Prevalence of MSI-H was low.

## Lay abstract

Certain biologic characteristics of tumors (or biomarkers) may be used to assess the likely course of a patient’s disease (i.e., their prognosis) and/or how they may respond to treatment. We evaluated whether the presence of the protein PD-L1 and high levels of microsatellite instability were associated with overall survival among patients with ten uncommon advanced cancers. PD-L1 was commonly expressed in solid tumors and its presence may be associated with shorter overall survival. Prevalence of high microsatellite instability was low.

The advent of immunotherapies targeting the PD-1 pathway has improved outcomes for patients with certain advanced solid tumors [1]. Checkpoint inhibitors targeting the PD-1/programmed death ligand 1 (PD-L1) immune checkpoint pathway block the interaction between PD-1 and its ligands, PD-L1 and PD-L2, and have demonstrated durable antitumor response and manageable toxicity among patients with a variety of solid tumor types [2–5]. PD-L1 is an immune checkpoint protein commonly expressed on tumor cells that interacts with PD-1 on T-cells and other immune cells, thereby inhibiting T-cell proliferation, cytokine production and cytotoxic activity [3,6]. Additionally, tumor cells may have defects in DNA mismatch repair (dMMR), which typically result in hundreds to thousands of somatic mutations, microsatellite instability (MSI) and presentation of potential neoantigens [7]. Tumors with high levels of MSI (MSI-H) upregulate immune checkpoint proteins, including PD-L1, enabling evasion of immune surveillance [8,9]. As response to anti-PD-1 therapies can vary from patient to patient and between tumor types [10], predictive biomarkers may identify patients more likely to benefit from such therapies [11]. Tumor PD-L1 expression has been established as a biomarker for patient selection for monotherapy with the anti-PD-1 monoclonal antibody pembrolizumab in multiple tumor types including advanced non-small-cell lung cancer (NSCLC), melanoma, cervical cancer, gastric cancer, head and neck squamous cell cancer and esophageal cancer [2,12–18], and MSI-H has been established as a tumor-agnostic biomarker for pembrolizumab monotherapy [2,19–21].

Activity of immune checkpoint inhibitors against uncommon solid tumor types is being evaluated in clinical trials. For example, KEYNOTE-158 is a single-arm, phase II, multicohort study investigating the antitumor activity and safety of pembrolizumab in ten advanced solid tumor types, regardless of biomarker status. Preliminary results from several of these cohorts demonstrated antitumor activity and durable responses, including in patients with PD-L1-positive or MSI-H tumors [17,22–25]. However, there are limited data on either the prevalence of PD-L1 positivity or MSI-H status in these tumor types. In addition, the potential relationships between these biomarkers and clinical outcomes among patients receiving standard of care (SOC) treatment (which for some patients included best supportive care) need to be assessed to provide context for interpreting results from studies without a comparator arm. We conducted a retrospective, observational study to evaluate the real-world prevalence of PD-L1 positivity (evaluated by immunohistochemistry [IHC]) and MSI-H status (evaluated by PCR or IHC) and the association of these biomarkers with overall survival (OS) among immunotherapy-naive patients who received SOC therapy in the same ten advanced tumor types included in the KEYNOTE-158 phase II multicohort study of pembrolizumab monotherapy.

## Methods

### Study design & patients

Eligible patients were identified within the Aarhus University (Denmark) pathology network using the Danish Pathology Register for tissue and the associated clinical database. The Danish Pathology Register is a nationwide data bank containing all clinical histology and cytology reports conducted by the country’s pathology departments [26]. Additional patient information was obtained through the Danish National Patient Registry. Eligible patients were ≥18 years of age at the time of diagnosis and had tissue samples collected between 1 January 2010 and 31 December 2015. However, because there were few cases with sufficient tissue availability for neuroendocrine tumors, biliary carcinoma and cervical carcinoma, allowance was granted to expand the study period to 1 January 2001 to 31 December 2015 for these three tumor types. Patients were included if they had a histologically or cytologically documented advanced (unresectable and/or metastatic) solid tumor of the following types: anal carcinoma, biliary adenocarcinoma (gallbladder or biliary tree [intrahepatic or extrahepatic cholangiocarcinoma] excluding Ampulla of Vater cancers), neuroendocrine tumors (including well-differentiated or moderately differentiated tumors of the lung, appendix, small intestine, colon, rectum or pancreas), endometrial carcinoma (excluding sarcomas and mesenchymal tumors), cervical carcinoma (including squamous cell carcinoma and adenocarcinoma), vulvar carcinoma, small-cell lung carcinoma (SCLC), mesothelioma, thyroid carcinoma and salivary gland carcinomas (excluding sarcomas and mesenchymal tumors). Patients were treated with SOC therapy, which was anticipated to comprise cytotoxic chemotherapy for most patients and for patients with poor performance status or no further documented treatment options, best supportive care. Given the study enrollment dates, patients were not anticipated to have received immunotherapy. Each formalin-fixed paraffin-embedded tissue was to produce up to 16 slides for biomarker analysis. Patients were excluded if they had an additional malignancy within 2 years of the cancer diagnoses under study. Between 40 and 45 patients were included for each of the cancer types of interest, with a preference for patients with samples collected most recently. This target was not met for neuroendocrine tumors (n = 30) or biliary carcinoma (n = 16) because of the availability of tissue samples of these cancers.

### Biomarker assessments

For biomarker analysis, slides were prepared from freshly cut samples at Aarhus University Hospital (Denmark) and then centrally evaluated by NeoGenomics Laboratories Inc. (Fort Myers, FL, USA). PD-L1 expression was assessed utilizing the PD-L1 IHC 22C3 pharmDx assay (Agilent Technologies, CA, USA); samples with a combined positive score (CPS) ≥1 were considered positive consistent with the prespecified PD-L1 CPS ≥1 cutoff point in the KEYNOTE-158 study. The CPS was calculated by summing the number of PD-L1-stained cells (tumor cells, lymphocytes, macrophages) and dividing the result by the total number of viable tumor cells, multiplied by 100 [27]. MSI was measured by PCR using the Promega MSI Analysis system version 1.2 (Promega Corporation, WI, USA) at one central laboratory (Almac Diagnostic Services, Craigavon, Northern Ireland). Samples were considered MSI-H if the PCR assay showed ≥2 of five MSI loci that differed in size from corresponding normal loci. For samples that could not be tested using the PCR assay, either because of the small amount of tissue or unavailability of a ‘normal’ control, IHC was performed at a single laboratory (Aarhus University Hospital). The sample was considered MSI-H if the IHC assay demonstrated loss of ≥1 of the proteins MLH1 (clone ES05, Dako, CA, USA), MSH2 (FE11, Dako), MSH6 (EP49, Dako) or PMS2 (EP51, Dako) (i.e., dMMR). PCR and IHC are commonly used in combination for MSI testing, and there is a very high correlation between PCR and IHC results [28].

### Statistical analysis

The frequency of biomarker expression (PD-L1 or MSI-H) was estimated with corresponding 95% CIs. OS analysis following first-line SOC included all eligible patients; an additional OS analysis was performed in patients who received only second-line therapy. OS (defined as the time from diagnosis [for first-line treatment], or from the start of second-line treatment, to death due to any cause) was evaluated by the Kaplan–Meier method, stratified by biomarker status. Log-rank tests were used to assess between-group differences in survival with a significance level set at 0.05. Cox proportional hazards models, both unadjusted and adjusted for age, sex, sample year, and Eastern Cooperative Oncology Group Performance Status (ECOG PS), were used to estimate hazard ratios (HR) and their associated 95% CIs. In addition to the primary analysis of outcomes evaluating a PD-L1 CPS ≥1 cutoff, a sensitivity analysis evaluating a PD-L1 CPS ≥10 cutoff was also performed.

## Results

### Patients

**Figure 1.**
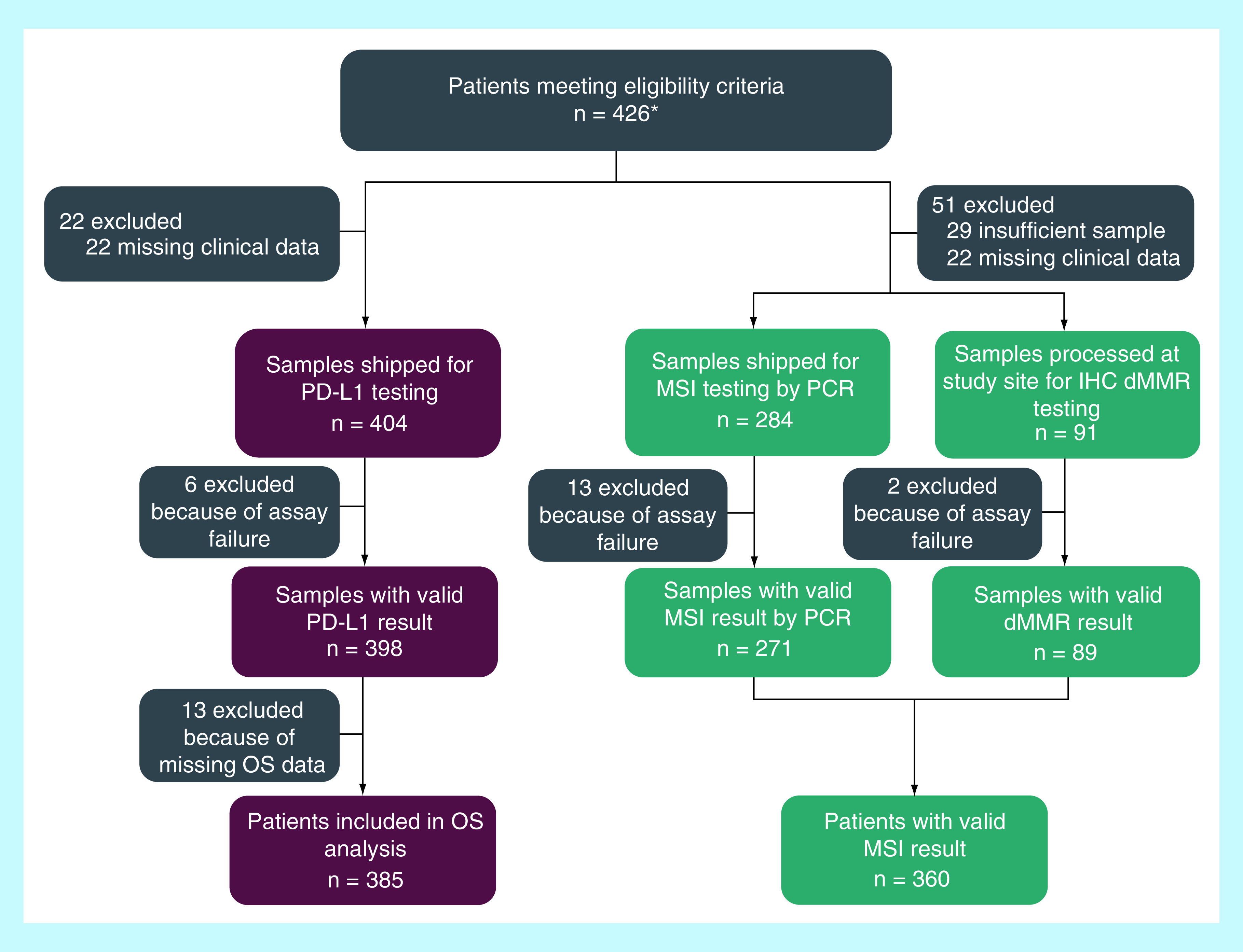
Patient selection and tumor sample evaluation of PD-L1 and microsatellite instability. Eligible patients were identified within the Aarhus University pathology network using the Danish Pathology Register for tissue and the associated clinical database. *Initial screening identified 435 patients; nine were excluded because of duplicate records. dMMR: Deficient mismatch repair; dMMR IHC: Immunohistochemistry deficient mismatch repair; MSI: Microsatellite instability; OS: Overall survival.

A total of 426 patients were identified from the Aarhus University pathology network and met eligibility criteria across the ten prespecified tumor types. Of these, 22 had insufficient or inadequate samples. For most of the tumor types, there were 40–49 samples, with the exception of neuroendocrine tumors (n = 30) and biliary cancers (n = 16). Baseline demographics and clinical characteristics are described in Table 1. Baseline characteristics among this population of patients who received SOC therapy between 2010 and 2015 (not anticipated to include immunotherapy) were generally similar between the PD-L1-positive and PD-L1-negative populations. Median age (PD-L1-positive, 67.0 years; PD-L1-negative, 62.0 years), proportion of female patients (PD-L1-positive, 65%; PD-L1-negative, 62%) and proportion of patients with ECOG PS of 0 (PD-L1-positive, 38%; PD-L1-negative, 37%) were similar between the groups. Among the 404 patients with adequate tissue samples evaluated for PD-L1 expression, six were excluded owing to assay failure and an additional 13 patients were excluded from OS analysis owing to missing critical clinical data; 385 patients were included in the OS analysis (Figure 1).

**Table 1.** Demographics and baseline characteristics of patient tumor samples.

Variables	All cancer types n = 404	Endometrial carcinoma n = 49	Thyroid carcinoma n = 46	Salivary glands n = 46	Small-cell lung cancer n = 45	Cervical carcinoma n = 44	Vulvar carcinoma n = 44	Mesothelioma n = 44	Anal carcinoma n = 40	Neuro-endocrine tumors n = 30	Biliary adenocarcinoma n = 16
Age, median (IQR), y	66 (64–67)	69 (63–71)	68 (61–72)	67 (60–70)	63.5 (62–70)	49 (44–54)	68 (66–73)	72.5 (69–76)	61 (56–64)	62 (60–67)	67 (62–76)
Sex, n (%)											
– Male	148 (36.6)	0	21 (45.7)	26 (56.5)	26 (57.8)	0	0	39 (88.6)	14 (35.0)	18 (60.0)	4 (25.0)
– Female	256 (63.4)	49 (100.0)	25 (54.3)	20 (43.5)	19 (42.2)	44 (100.0)	44 (100.0)	5 (11.4)	26 (65.0)	12 (40.0)	12 (75.0)
Year sample collected, n (%)											
– 2000 or earlier	7 (1.7)	0	2 (4.3)	0	0	2 (4.5)	1 (2.3)	0	2 (5.0)	0	0
– 2001–2009	126 (31.2)	1 (2.0)	17 (37.0)	28 (60.9)	18 (40.0)	23 (52.3)	9 (20.5)	0	19 (47.5)	6 (20.0)	5 (31.3)
– 2010–2015	271 (67.1)	48 (98.0)	27 (58.7)	18 (39.1)	27 (60.0)	19 (43.2)	34 (77.3)	44 (100.0)	19 (47.5)	24 (80.0)	11 (68.8)
ECOG PS, n (%)											
– 0	151 (37.4)	24 (49.0)	27 (58.7)	17 (37.0)	6 (13.3)	29 (65.9)	19 (43.2)	12 (27.3)	10 (25.0)	2 (6.7)	5 (31.3)
– 1	87 (21.5)	8 (16.3)	8 (17.4)	2 (4.3)	15 (33.3)	10 (22.7)	12 (27.3)	13 (29.5)	7 (17.5)	6 (20.0)	6 (37.5)
– 2+	38 (9.4)	2 (4.1)	4 (8.7)	2 (4.3)	11 (24.4)	0	1 (2.3)	14 (31.8)	3 (7.5)	0	1 (6.3)
Missing/unknown	128 (31.7)	15 (30.6)	7 (15.2)	25 (54.3)	13 (28.9)	5 (11.4)	12 (27.3)	5 (11.4)	20 (50.0)	22 (73.3)	4 (25.0)

ECOG PS: Eastern Cooperative Oncology Group performance status; IQR: Interquartile range.

Of the 426 patients meeting eligibility criteria, 51 were excluded from MSI evaluation due to insufficient or inadequate sample. 284 samples were submitted for MSI PCR testing; 13 samples were excluded because of assay failure. The remaining 91 samples, which did not contain sufficient ‘normal tissue’ for PCR using the Promega MSI Analysis system, were tested with an IHC-based assay targeting the proteins involved in the mismatch repair system. Of the 91 samples processed at the study site for dMMR IHC testing, two were excluded because of assay failure. Overall, 360 patient samples had a valid test for mismatch repair deficiency (MSI assay, n = 271 and dMMR IHC, n = 89) (Figure 1).

### Prevalence of PD-L1 expression

**Figure 2.**
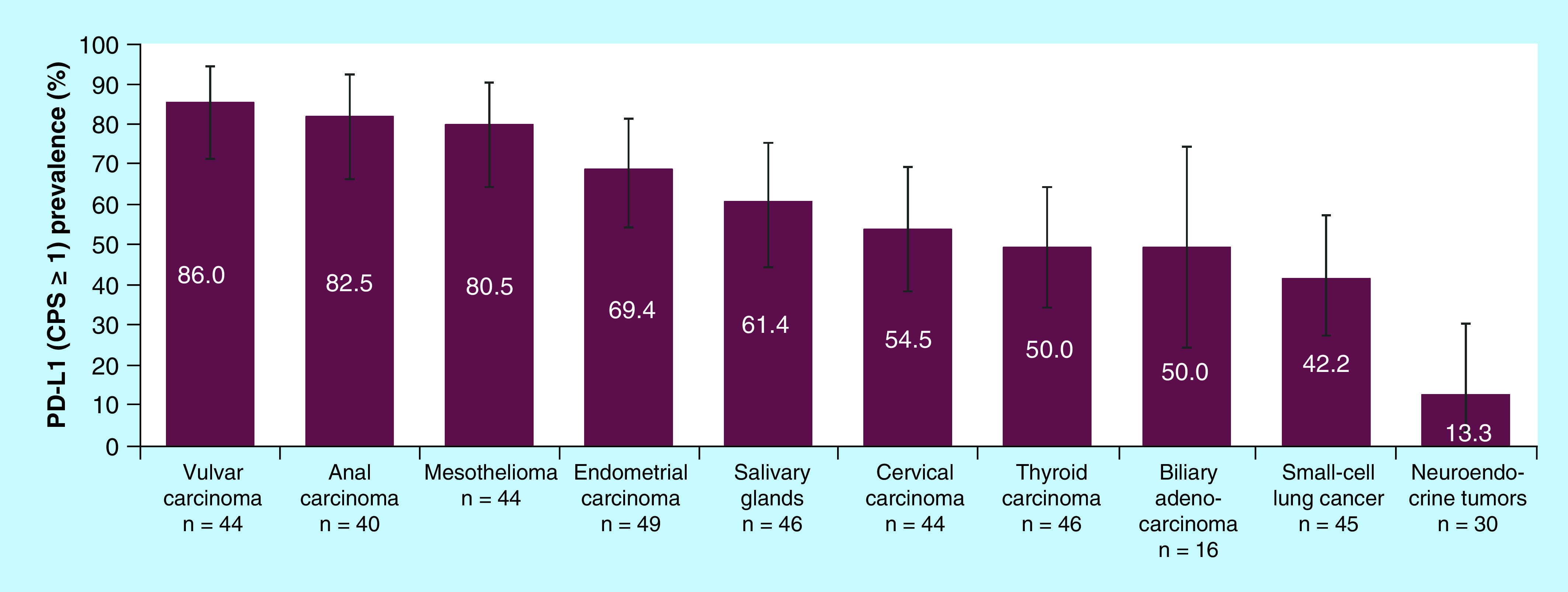
Prevalence of PD-L1 combined positive score ≥1 by tumor type. Analysis included all tumor types. Evaluation of PD-L1 CPS in tissue samples was performed using the PD-L1 IHC 22C3 pharmDx assay (Agilent, CA, USA) and samples with a CPS ≥1 were considered positive. IHC: Immunohistochemistry; PD-L1 CPS: PD-L1 combined positive score.

Among the 398 patients with valid PD-L1 IHC results, 242 (61%; 95% CI: 56–66%) had PD-L1 CPS ≥1. PD-L1 expression was detected in all tumor types evaluated. With the exception of small-cell lung cancer (42%; 95% CI: 28–58%) and neuroendocrine tumors (13%; 95% CI: 4–31%; Figure 2), the prevalence of PD-L1 expression (i.e., CPS ≥1) in each of the tumor types evaluated was greater than 50%. The distribution of PD-L1 positivity was similar in the total study population and among patients who received second-line treatment (39 and 36%, respectively).

### OS & PD-L1 expression

**Figure 3.**
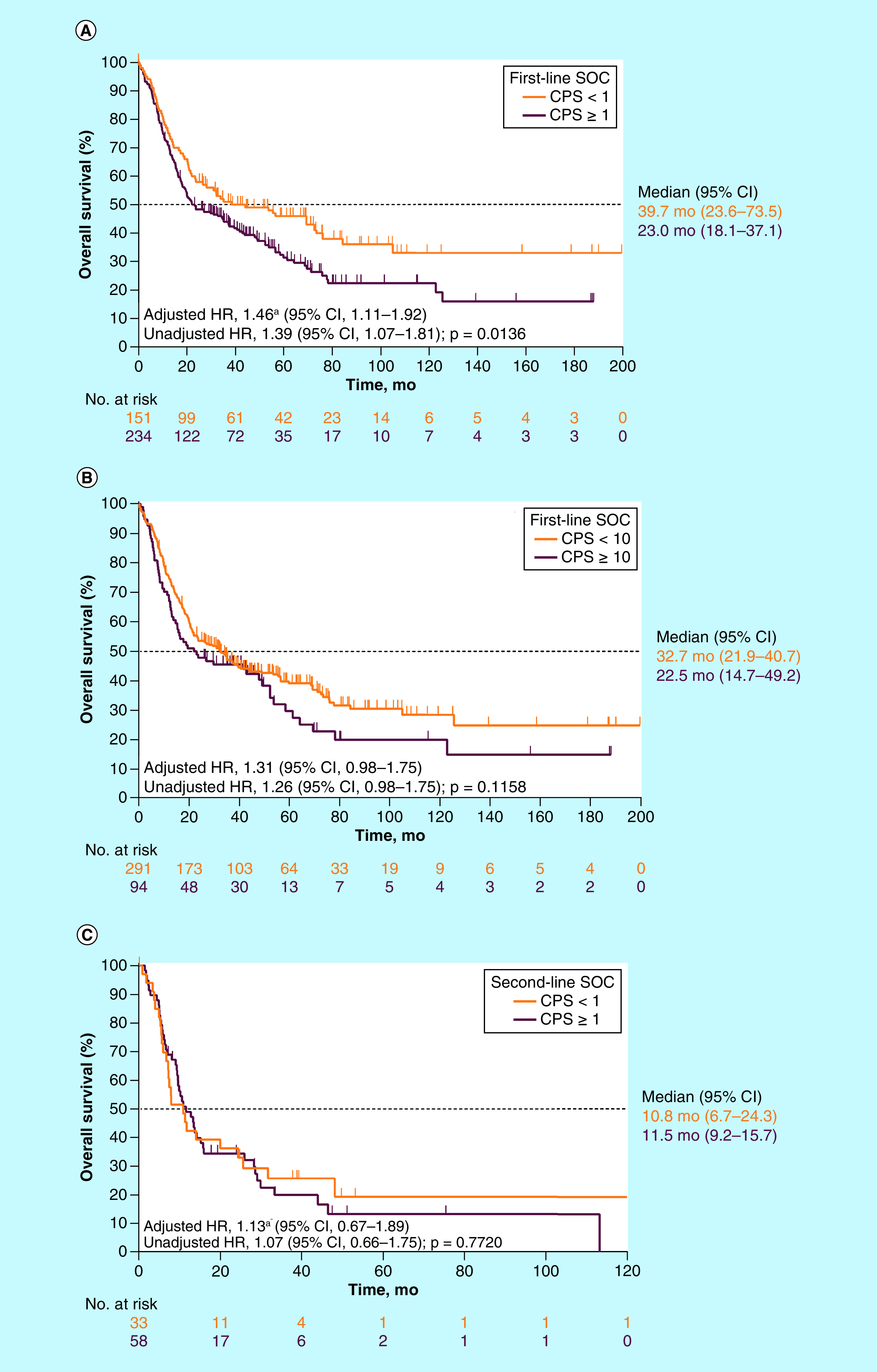
Overall survival by PD-L1 combined positive score. Kaplan–Meier estimates of overall survival among patients with **(A)** PD-L1 CPS ≥1 and <1 in the overall study population after first-line treatment, **(B)** PD-L1 CPS ≥10 and <10 in the overall study population after first-line treatment, and **(C)** PD-L1 CPS ≥1 and <1 in the overall study population after second-line treatment. ^a^Adjusted HR is based on Cox proportional hazard models that included year (derived from sample collection date), age, sex, and ECOG. Missing ECOG PS was treated as a separate category and included in the model for adjustment. Overall survival was defined as the time from diagnosis to death from any cause. CPS: Combined positive score; ECOG PS: Eastern Cooperative Oncology Group performance status; HR: Hazard ratio; Mo: Months; SOC: Standard of care.

OS after the start of first-line SOC therapy was significantly shorter in patients with PD-L1 CPS ≥1 (n = 242) than in those with PD-L1 CPS <1 (n = 156): median OS was 23.0 months in patients with CPS ≥1 (95% CI: 18.1–37.1) and 39.7 months in patients with CPS <1 (95% CI: 23.6–73.5) with an unadjusted HR of 1.39 (95% CI: 1.07–1.81; p = 0.0136; Figure 3A). A similar outcome was observed in analyses adjusted for age, sex, sample year and ECOG PS (HR: 1.46; 95% CI: 1.11–1.92). Because these adjustments did not meaningfully alter the outcome and because the adjusted analyses excluded a significant proportion of patients owing to missing ECOG PS data, we subsequently focused on unadjusted analyses. A similar trend was observed when the cut point was set at PD-L1 CPS ≥10 versus CPS <10. Median OS was 32.7 months in patients with PD-L1 CPS <10 (95% CI: 21.9–40.7) and 22.5 months in patients with CPS ≥10 (95% CI: 14.7–49.2), with an unadjusted HR of 1.26 (95% CI: 0.98–1.75; p = 0.1158; Figure 3B). Similar findings were observed with PD-L1 CPS ≥50 as the cutoff. The HR for OS was 1.29 (95% CI: 0.81–2.04) for CPS ≥50 versus CPS <50. However, there were only 27 patients with PD-L1 CPS ≥50.

OS after initiation of second-line (nonimmunotherapy) treatment (n = 91) did not differ significantly by PD-L1 status (unadjusted HR: 1.07 [95% CI: 0.66–1.75]; p = 0.77). Median OS was 10.8 months in patients with a CPS <1 (n = 33; 95% CI: 6.7–24.3) and 11.5 months in patients with CPS ≥1 (n = 58; 95% CI: 9.2–15.7; Figure 3C).

Similar findings were also observed in a sensitivity analysis that defined OS from the time of second-line treatment initiation to death from any cause (p = 0.23). Median OS was 10.8 months among patients with a CPS <10 (n = 72; 95% CI: 7.9–15.1) and 13.4 months in those with CPS ≥10 (n = 19; 95% CI: 6.1–44.0), resulting in an unadjusted HR of 0.92 (95% CI: 0.25–1.63; p = 0.77).

### Prevalence of MSI-H

MSI-H was detected in only eight (2.2%; 95% CI: 0.9–4.3%) of the 360 evaluable tumor samples. All MSI-H tumors identified were in patients with gynecologic cancers, including endometrial carcinoma (7/49 [14.3%; 95% CI: 6.0–27.3%]) and cervical carcinoma (1/44 [2.3%; 95% CI: 0.1–12.1%]; Figure 2). Among the eight patients assessed as MSI-H, seven (87.5%) were also PD-L1 positive. Clinical outcomes by MSI-H status were not evaluated due to the low prevalence of MSI-H tumors.

## Discussion

This retrospective longitudinal study evaluated the prevalence of PD-L1 expression (as assessed by CPS ≥1) and identified an association of tumor PD-L1 (CPS ≥1) expression with shorter OS in response to first-line SOC therapy in patients with ten prespecified solid tumor types. Because patients from 2010 to 2015 were included, patients were anticipated to be immunotherapy-naive. Expression of PD-L1 by IHC was detected in 61% of evaluable tumor samples overall; the prevalence varied by tumor type (range: 13–87%). Notably, PD-L1 expression was detected in all evaluated tumor types, with prevalence ranging from 13% (neuroendocrine tumors) to 86% (vulvar carcinoma). PD-L1 CPS ≥1 was associated with shorter OS after first-line treatment compared with PD-L1 CPS <1, whereas PD-L1 CPS ≥10 was not associated with shorter OS after first-line treatment. There was no significant difference in OS by PD-L1 status after second-line treatment. Overall, these results demonstrate that PD-L1 is commonly expressed in a variety of advanced solid tumors, with a prevalence of ≥50% in eight of the ten tumor types evaluated, and that PD-L1 expression is not associated with improvement in OS after either first-line or second-line SOC therapy.

Evidence from prospective clinical studies of pembrolizumab monotherapy has indicated an association between higher tumor PD-L1 expression and improved clinical outcomes [12,29,30]. However, there are limited data evaluating any potential prognostic value of tumor PD-L1 expression [30] in patients receiving SOC therapy (i.e., nonimmunotherapeutic treatments, particularly those with less frequently occurring tumor types such as those included in this study). Results from the current retrospective longitudinal study suggest that PD-L1 expression is associated with shorter OS after first-line SOC therapy (nonimmunotherapy) across a range of tumors (unadjusted HR: 1.39 [95% CI: 1.07–1.81]; p = 0.0136). Consistent with our findings, previous studies have identified an association of PD-L1 expression with shorter OS in patients with malignant salivary gland tumor [31], malignant pleural mesothelioma [32], NSCLC [30,33] and pancreatic cancer [30,34]. In contrast, other studies have reported that PD-L1 expression was associated with longer OS in patients with NSCLC [35] and SCLC [36]. Furthermore, other studies have reported no prognostic value for PD-L1 expression in patients with cervical cancer [37], SCLC [33] and anal squamous cell carcinoma [38]. A number of factors may have contributed to these differences, including but not limited to differences in PD-L1 assessment techniques and small-sample sizes. Additionally, it is important to note in a number of the aforementioned previous reports, it was not clearly defined whether patients had received or were receiving treatment, and this may have confounded assessment of the prognostic value of PD-L1 expression. Furthermore, because the tumor microenvironment is made up of heterogeneous cell populations, other biologic processes (potentially including but not limited to: secretion of growth factors and chemokines, production of blood vessels, myeloid-derived suppressor cell production of nitric oxide synthase and reactive oxygen species and infiltration of T-regulatory cells) may influence the effectiveness of PD-L1 as a biomarker among different tumor types [39]. At present, it is uncertain how such processes might influence the prognostic value of PD-L1.

The findings from the current study may aid in the interpretation of single-arm studies where it can be difficult to evaluate whether a biomarker is truly predictive of treatment outcome or is prognostic (i.e., indicative of outcome irrespective of treatment). Our results suggest that tumor PD-L1 CPS is not a positive prognostic factor for OS in patients with ten uncommon tumor types who received SOC therapy. As such, when evaluating single-arm trial data for checkpoint inhibitors (including pembrolizumab studies such as KEYNOTE-158), it is likely that any association between tumor PD-L1 CPS and a favorable outcome is representative of predictive value rather than of selection of patients with improved prognosis.

In our study, the overall prevalence of patients with MSI-H tumors was only 2.2%, with seven out of eight MSI-H tumors occurring in the endometrial cancer group in which the prevalence of MSI-H was 14.3% (95% CI: 6.0–27.3%). These findings suggest lower prevalence of MSI-H where earlier studies have reported MSI-H frequencies between 28.3 and 31.37% in endometrial cancer [40,41]. This may potentially be explained by better prognosis for patients with MSI-H who would be underrepresented in this study, which focuses on patients with advanced/incurable disease. The KEYNOTE-158 study previously demonstrated the clinical benefit of pembrolizumab among patients with previously treated unresectable or metastatic MSI-H/dMMR noncolorectal cancer, providing further evidence to support MSI-H as a predictive biomarker for response to anti-PD-1 therapy [25].

This study is one of the largest to investigate associations between PD-L1 and OS in patients with uncommon cancers receiving SOC therapy. All biomarker assessments were conducted in a standardized fashion within a single central laboratory to minimize variability in evaluation. Additionally, patients were identified through a nationally representative network in Denmark instead of a single center. However, despite the large overall sample size, the numbers of patients in each individual cancer type did not allow further by-cancer-type analyses. An important confounding factor in our retrospective analyses was that we compared OS in a mixed and heterogeneous population of patients with different proportions of tumor types and baseline characteristics between the two groups.

## Conclusions

PD-L1 is commonly expressed in a range of advanced tumors, and we demonstrate an association of tumor PD-L1 expression with shorter OS in patients after first-line SOC therapy.

## Future perspective

Results from this study may be used to aid in interpretation of clinical trials evaluating PD-L1 as a predictive biomarker for pembrolizumab therapy. Prospective studies may further delineate the prognostic value of PD-L1 expression and MSI-H in the tumor types evaluated.

**Summary points**

- This is one of the largest studies to investigate associations between PD-L1 expression (by combined positive score) and overall survival in patients with uncommon cancers (anal, cervical, endometrial, salivary gland, small-cell lung, thyroid and vulvar carcinoma, biliary adenocarcinoma, mesothelioma and neuroendocrine tumors) receiving standard-of-care therapy, including best supportive care.
- PD-L1 and high levels of microsatellite instability are established biomarkers for PD-1/PD-L1 immune checkpoint inhibitors, but current evidence for the prognostic value of PD-L1 is limited.
- PD-L1 was expressed in all ten advanced uncommon cancers with an overall prevalence of 61%.
- We report an association of tumor PD-L1 expression (combined positive score ≥1) with shorter overall survival in patients after first-line standard-of-care therapy, including best supportive care.
- There was no association between tumor PD-L1 expression and overall survival after second-line therapy.
- High level of microsatellite instability expression was identified infrequently and only in endometrial and cervical carcinomas.
